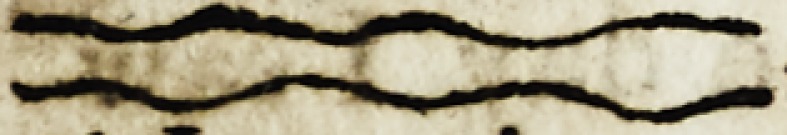# On Chronic Peritonitis, with a Case and Dissection

**Published:** 1815-07

**Authors:** J. K. Walker

**Affiliations:** Physician to the Huddersfield Dispensary.


*For the Medical and Physical Journal.*

### *On Chronic Peritonitis, with a Case and Dissection;* by J. K. WALKER, M. D.

FROM several opportunities which I have had of examining morbid structure, and particularly from the history of the case which forms the prominent feature of the present present paper, I am induced to submit to your readers the conclusion which I have formed—that the occurrence of chronic peritonitis is far more frequent than the accounts of medical writers would authorize us to believe. From its truly chronic state—from the obscurity of its development—from its consecutive complication with other disorders—and from the occasional difficulty of discriminating, in the mass of symptoms, those which mark the primary affection, I am of opinion that many symptoms have been laid to the account of other organs, which owe their rise to this. Dr. Pemberton, in his able Disquisition (on the various Diseases of the Abdominal Viscera), is the first author, I apprehend, who has treated this subject in a separate discussion; and the reader will there find the authorities of Lommius and Fernelius, quoted in attestation of the multiform and insidious aspect of the disease. From the slight tension of the skin that occurs, from the long remissions of pain, from the kind of co-existent convalescence which the patient seems to enjoy, the latent mischief may elude detection for years, until rueful experience shall develope to the sufferer the awful truth—

Hæret lateri lethalis Arundo.

In its inchoate state it will seldom offer much impediment to the usual avocations; perhaps, a little soreness in one or more parts of the abdomen is all that the patient has to complain of, and, if any irregularity in the bowels exists, the removal of it is often a temporary alleviation. This is one cause, I apprehend, of a pseudo-ascription of the disease to a fault of the alimentary canal, which is a mere secondary or consecutive affection. Doubtless, the convolutions of the intestines may, in time, undergo accretion from adhesive inflammation: the peripheral surface of the liver, the omentum, mesentery, or all the abdominal viscera, may successively participate in the affection. It may be, that the pathognomonic symptoms of each or all of the diseases of these parts, may alternately present themselves, so as to elude the precision of the acutest examiner, yet shall the origo malorum all flow from antecedent chronic inflammation of the peritonæal membrane. The inflammation of other abdominal viscera may superinduce chronic peritonitis, and thus they may come in time to be complicated; but the idiopathic form I here wish to describe, is not necessarily attended with symptomatic fever, or intensity of pain, and its combination with other diseases is only incidental. It has been broached, indeed, that the morbific matter may be translated without being removed from the habit, and that the disorder will thus recur at intervals, longer or shorter, according to the idiosyncrasy of the individual. I have no authority at hand to to contravert this position, but, if it imply also the possibility of its entire conversion into another disease, some bad consequences, I should think, would be apt to ensue from a total oversight of the first exciting cause. The late illustrious Dr. Ferriar, the principal advocate of the doctrine of the conversion of diseases, did never, I am persuaded, intend by it to sanction the impossibility of the recurrence of the first disease, however long it might be suspended. The mesentery, which is a mere duplicature of the peritonæum, may be first inflamed, and afterwards the peritonæum which lines the parietes of the abdomen. If there be any line of demarcation between the two affections, the chylous appearance of the stools in one of them will form the discriminating feature. Sennertus, speaking of mesenteric inflammation, alludes to the permutation and suspension of this disease in the following remark: "Sæpe vero materia a naturâ alio transvertitur, non tamen plane tollitur; unde malum recrudescit, et sæpe per multos annos, imo totum vitæ tempus durat, nunc febre redeunte, nunc eadem in colicos dolores permutata, ac cessante febre singulis mensibus, vel alio intervallo colica redeunte, et si non inflammatione, tamen φλογωσει excituta."

I have been told by a medical friend, who witnessed some dissections of patients who had died of puerperal fever, that, in one of the cases which was fatal in less than a week after delivery, very extensive ulceration of the omentum and mesentery were found, the first rudiments of which must have existed, he thinks, anterior to parturition.* Could the disease, in this case, have derived its fomes from absorption of purulent sanies? Chronic peritonitis, however, the present object of discussion, will frequently arise without any assignable cause. I suppose the usual sources (familiar to all medical men) that excite inflammation in analogous membranes are equipollent in this. In the case here cited more at large, the patient ascribed his ailments originally to a habit of immoderate exercise of the body and subsequently cooling too rapidly. It was always aggravated by a sluggish state of the bowels. The diagnosis, I apprehend, is never difficult, until the supervention of consecu-

* It would have enhanced the value of Mr. William Hey's Treatise on Puerperal Fever, if, at the time that he had advocated the opinions and treatment of Dr. Gordon, he had imitated him likewise in subjoining some account of the morbid appearances observed on dissection, as it would seem, before the practice of that eminent physician was resorted to, ample opportunity was afforded him of obtaining that desideratum.

tive tive disease masks the original one. Where the affection is isolated and vigilantly treated from its infancy, the efforts of nature and art will produce, in some cases, an auspicious crisis; in others, a mere truce to the disorder. We know, by fatal experience, that the issue of a relapse is often disastrous—a transition to a more acute form, or to a disease of a still more inflexible nature, are ominous foreboders of a fatal catastrophe. The morbid appearances are such as Dr. Baillie has described them to be, a thicker state of the peritonæum, which is opaque and pulpy, without any extension of the inflammation to the muscles of the abdomen; whatever other morbid appearances are found exterior to the peritonæum are not essential to the primitive affection, and are only to be regarded as accidental, from the extension and increased violence of the symptoms of the first disorder, or from the supervention of or conjunction with a secondary one. In an elderly woman, who had been formerly relieved by Mr. Parkinson, surgeon, of Leicester, and myself, in some anomalous affection of the abdominal viscera, and who afterwards died of abdominal dropsy, we discovered very extensive adhesions and broad surfaces of thickened peritonæum, yielding implicit evidence of pre-existing inflammation. She owed it, I believe, to the vigilant attention of her surgeon, that she did not earlier experience the dismal effects of this dangerous affection.

For the M. M. I have nothing new to offer, but would strenuously urge the adoption of vigorous treatment in its nascent state. The treatment of Dr. Pemberton is the most judicious that could be adopted. Perhaps, the more liberal use of the warm bath, warm fomentations, tepid lavements, and the institution of permanent drains, might prove useful auxiliaries. Mr. Raussier, who attempts to prove that what we call peritonitis is of a catarrhal nature, declares that he has had success from the vapour bath and mercurial friction, but of these I cannot speak from experience.

The very extensive scene of disease which presented itself on examining the body of William Turner, a patient in the Huddersfield Dispensary, and the violence of the transition from a latent to an active state of peritoneal affection, gave me an interest in reciting some of the most prominent features of his disease. In the vigour of life (æt. 45,) until three or four months back, he made no complaint, and about that time, I understand, he felt occasional uneasiness in different parts of the abdomen, but chiefly on the right side; it was only now and then that pressure gave him pain. He ascribed his symptoms to a sluggish state of the bowels, and the relief which he always, for a time, received from aperients, rients, countenanced him in his opinion. Though the exercise of his ordinary avocations was not precluded, yet the least irregularity, or extraordinary exertion, either of mind or body, always reminded him of the latent mischief. From the extension of the affection to other abdominal viscera, a multitude of phenomena arose, which would have rendered it difficult for a person, without a knowledge of the preceding symptoms, to have pointed out the original affection. His retention of bulk, however, proved to me that the lacteals were not bereft of their functions; for, had any very material protracted affection of the small intestines existed, some emaciation must have supervened. The same might be said generally of the other chylopoietic viscera, for, though the peripheral surface of the liver was connected by adhesions, and its enlarged volume seemed a little to impede respiration, yet neither the gall bladder, duodenum, or stomach, were in the least implicated. On the 17th of April, the day when the symptoms of acute peritonitis began to demonstrate themselves in company with other indications of more extended disease, immediate recourse was very properly had by my friend Mr. Wilkes, who attended him, to repeated copious abstraction of blood, which was slightly sizy but not cupped. The application of leeches just over the cœcum (where he described his pain to be greatest) and warm fomentation gave but slight relief. On the day I saw him (20th of April) the congeries of symptoms, dyspnœa, short dry cough, nausea, obstinate costiveness, increased abdominal tension and tenderness, convinced me that the inflammation had extended its ravages beyond all redemption. From the first he had taken, every three or four hours, cathartic mixtures and boluses, from all which he had but one mere watery dejection on the third day, without any of the natural properties. Cathartic lavements variously medicated, first to a pint and afterwards to a quart every four hours, were administered, but always returned unaltered. He used the last two days the following tobacco glyster, with the same want of success and without any nausea:

℞. Tabaci Folior, ℥ii.
Aquæ Ferventis, Oij.
Macera per horam in vase leviter clauso et cola, subinde Colaturæ adde Extracti Colocynthidis composit. ℨſs. Misce ft. Enema singulis quartis horis injiciendum.

Since the leeches were last applied, he had tried three large blisters successively in different parts of the abdomen. The symptoms, however, on the fifth and sixth day, afforded more unequivocal indicia of approaching dissolution. His pulse became weaker and more irregular. Great restlessness, alternate vigilia and delirium preceded the fatal catastrophe, which took place on the eighth day of this last attack. His countenance, during the last days, was more than usually red and bloated, or what the French physicians would term *vulteuse.*

On inspecting the body along with Messrs. Wilkes and Robinson, surgeons, we observed that nearly the whole of the peritonæum had been subject to inflammation, both that which covers the parietes of the abdomen, and great part of that which forms the exterior coat of the intestines and the liver; it was thicker than natural in many parts, in others coated with coagulable lymph. Large tracts of the abdominal peritonæum also were in a state of ulceration, exhibiting to the eye the traces of the subjacent intestines in a state of ulceration also. Throughout the intestinal tube, from the end of the duodenum to the rectum, there were not two inches together free from marks of inflammation and ulceration in the peritoneal coat, without any corresponding change, however, of the villous one. They were agglutinated by adhesive inflammation to each other as well with the neighbouring viscera. The intestinal cylinder, in many parts, was alternately dilated and constricted in this manner , or, as Morgagni would have explained it, "Intestina valde distenta et alibi quasi filo constricta." There was more than a quart of fluid in the cavity of the abdomen, partly serous, partly puriform, of a cineritious colour, and of a very offensive odour. The mesentery and omentum were equally affected with the above viscera, and the liver was augmented in bulk, and of a deep purple internally.

If I had not encroached too long already on the patience of your readers, I could adduce other instances, on my own testimony, and on the authority of the ablest practitioners, in proof of the insidious properties of this disease, its tendency to transformation and sudden transition to a more complicated and fatal form. But, whoever has studiously perused the writings of Morgagni, Baillie, Sœmering, and others who have treated on the organic changes brought on by disease, must have remarked, what a vast mass of affections there are, of which there are not only no pathognomonic symptoms, but scarcely any intelligible clue to guide us to the real affection. He must have felt the futility and folly of adhering to any system in a science avowedly conjectural, in which there is little more than a choice of presumptions. Every future improvement in the diagnostic branch of medicine dicine must arise from observations drawn from morbid appearances as well as clinical observation. From such authors as Dr. Baillie and Dr. Farre, this part of our science has lately derived a great accession of improvement, and may still expect to reap more. In science in general, we are willing to concede our admiration to him who shall be successful in devising the most ingenious and plausible theory, but in the medical art, it is to the man, who, contemning the seductive glare of hypothesis, and extricating himself from its trammels, shall, through labours and patient researches, add one useful fact more than those which were already known, that the palm of public applause is due. It is such authors as these, that the profession is taught to venerate and look up to, as containing a fund of knowledge and experience which no other source can impart. They will (if I may, in part, adopt the language of an elegant writer*) become the medical patriarchs of future days; as long as their works shall live, they will never be dead to society; they will have niches in the temple of learning which nothing but oblivion can destroy. Medical systems are ephemeral and will die away, but facts are imperishable; they are not to be blown away by the blowing of the breath of displeasure, like dust upon the shelf; but will remain a ponderous mass of accumulated wisdom, the solidity of which will survive the wreck of the most celebrated and brilliant hypothesis. Whatever revolutions may befall the current dogmas of our days in ages to come, honour will continue to be paid to the memory of such as these for their accuracy and professional zeal, as long as genius and philanthropy are esteemed among mankind.

J. K. WALKER, M. D.
*Physician to the Huddersfield Dispensary.*

---

*For the Medical and Physical Journal.*

### Londinensis *in Reply to* Mr. Smerdon.

* Dr. Yeats.

## Figures and Tables

**Figure f1:**